# Soil applied potassium improves productivity and fiber quality of cotton cultivars grown on potassium deficient soils

**DOI:** 10.1371/journal.pone.0250713

**Published:** 2021-04-29

**Authors:** Shabir Hussain, Hakoomat Ali, Syed Tahir Raza Gardezi

**Affiliations:** Department of Agronomy, Faculty of Agricultural Sciences and Technology, Bahauddin Zakariya University, Multan, Pakistan; Harran Üniversitesi, TURKEY

## Abstract

Cotton (*Gossypium hirsutum* L.) is considered as the most valuable cash crop of Pakistan. During last decade, its yield has been declined due to various biotic and abiotic factors. Among abiotic factors, improper use of fertilizers is considered very important specially regarding plant defense and yield. This study was conducted to evaluate the effect of different levels (0, 40, 80 and 120 kg ha^-1^) of K fertilizer (K_2_O) on different growth parameters of two commercial Bt cotton cultivars (CYTO-301 and IUB-2013) and one non-Bt cultivar (CYTO-142) during 2016 and 2017. Maximum plant height (124–134 cm), dry matter contents (915–1005%), fruiting point (441–462), bolls per plant (96–139), average boll weight (4.2–5.2 g) and seed cotton yield (2524–3175 kg ha^-1^) and minimum shedding (43–73%) were observed in plots receiving highest dose of K (120 kg ha^-1^). The CYTO-103 cultivar was found more responsive to K fertilizer as compared to rest of cultivars (CYTO-142 and IUB-2013). Concluding, ideal dose of fertilizer is very important (120 kg ha^-1^ in our case) for optimum growth and production of good quality fiber with enhanced seed cotton yield.

## Introduction

Cotton (*Gossypium hirsutum* L.) is commonly termed as “White Gold” owing to its quality fiber and its adaptability to various ecological zones across the world [1]. It is cultivated mainly for its fiber and low cholesterol oil. It also plays a key role in the economic and social affairs of the world providing basic inputs to the textile industry [2, 3]. It is considered as the backbone of Pakistan’s economy. Area under cultivation of cotton is around 2.373 million hectares with annual production of 9.861 million bales. It has major share in gross domestic product (0.8%) and agriculture value addition (4.5%) [4]. Pakistan is among the top leading cotton producing countries of the world [5].

During last decade, per unit area yield of cotton is decreasing due to various biotic and abiotic factors. Among various abiotic stressors, imbalance use of fertilizers has received great attention of researchers [6, 7]. Optimum use of fertilizers in any crop is crucial for obtaining better crop yields and economic returns (Pettigrew *et al*. 2005). Nutrition application is considered one of the major factors for increasing yield on a per hectare basis [8].

Along with other macro elements, well-balanced potassium nutrition is important for producing a high quality, high-yielding cotton crop [9]. Among synthetic fertilizers, the use of potassium (K) fertilizers is quite low in cotton, especially in Pakistan [10]. Severe K deficiency in cotton can decrease yield and reduce fiber quality by decreasing the expansion of leaf area and CO_2_ assimilation capacity [11, 12]. Fast-growing and early maturing cultivars are more sensitive to K-deficiency [13]. Indirect indicators of K-deficiency may include early wilting, variable yields, early senescence and poor quality [14].

The soil application of macronutrients (including K) is the most common way of improving soil fertility [15]. However, high doses of fertilizers are needed for soil application [16]. Macronutrients (particularly K) can fix within the soil depending upon the charge of clay minerals; thus, reducing their availability to the crops [17].

Utilization of mineral fertilizers is sought as an effective strategy to improve soil nutrient and boost cotton yield [18]. Though the role of K is well established in the cotton [19], but a study on appropriate dose of K in combination with the commercial available and genetically different cultivars was lacking according to ecological conditions of Pakistan. Keeping in view the above mentioned facts, the present study was conducted to evaluate the response of different cotton cultivars under different K levels and their effect on productivity, seed-cotton yield and fiber quality grown under the agro-ecological conditions of Multan, Pakistan.

## Materials and methods

### Experimental particulars

A two years (2016–2017) field study was conducted to evaluate the influence of different levels of K (K) fertilizer on growth, productivity and fiber quality of cotton cultivars at the Research farm of Central Cotton Research Institute, Multan, Pakistan. Before sowing of the crop, soil samples were collected from experimental field to a depth of 30 cm and soil was found K deficient. All other physical and chemical parameters of soil, collected from experimental field are shown in Table 1. For experiment, two Bt cotton cultivars (CYTO-301 and IUB-2013) and one non-Bt cultivar (CYTO-142) were used. The CYTO-301 and CYTO-142 were developed by Central Cotton Research Institute, Multan and IUB-2013 was developed by the Islamia University, Bahawalpur, Pakistan. The three cultivars were sown in the main plots while four fertilizer doses (0, 40, 80 and 120 kg ha^-1^ of K_2_O) were applied to the sub plots. A randomized complete block design (RCBD) with split plot arrangement with three replications was selected for the study.

**Table 1:** Physico-chemical analysis of experimental soil (on dry weight basis).

Parameters	2016	2017
**A. Mechanical Analysis of Soil**		
Sand (%)	19	22
Silt (%)	41	40
Clay (%)	40	38
**B. Chemical Analysis of Soil**		
EC (d Sm^-1^)	1.79	1.81
pH	8.01	8.02
Organic matter (%)	0.66	0.63
Total nitrogen (%)	0.041	0.040
Available P (ppm)	5.52	5.58
Available K (ppm)	99	98

### Crop husbandry

Seedbed was prepared by three cultivations of a tractor mounted cultivator followed by planking. The sowing was done manually with single row cotton drill in 75 cm apart rows where seed rate was 20 kg ha^-1^. In order to adjust recommended plant population, thinning was done after 28 days of sowing by pulling out the extra plants manually. Plant-to-plant distance was adjusted up to 25 cm. Seeds were delinted with concentrated H_2_SO_4_ before sowing. The recommended doses of nitrogen and phosphorus fertilizers i.e. 250 kg ha^-1^ and 100 kg ha^-1^ were used as per standard practices. Weed control and recommended plant protection measures were followed for the control of sucking pests and bollworms. The calculated amount of irrigation water was applied to each experimental unit at regular intervals depending upon the climatic conditions. The experimental crop was harvested in the 2nd week of November during 2016 and 2017. Except the experimental treatments, all other agronomic practices like irrigation, weeding, and insect pest control were applied uniformly in all experimental units.

### Growth and yield parameters

At maturity, the height of five randomly selected plants from each replication was measured from the base of the plant to the tip of the main stem with measuring tape. Similarly, five plants were chosen randomly after harvesting for estimation of plant dry matter. The selected plants were sun dried and then weighed individually. Twenty plants were chosen randomly and tagged in every plot and total fruiting points were counted manually. Finally means were computed for each replication. Shedding points were also recorded randomly selected 20 plants and shedding percentage was assessed from each experimental unit followed by computing means. Mature and effective bolls were picked and counted from the randomly selected plants at maturity and were averaged to calculate the number of bolls per plant. For the average boll weight (g) data 20 effective and opened bolls were selected at random from each treatment. They were separated from plants and were weighed along with locules. The average boll weight was computed and expressed in grams (g). The seed cotton yield per hectare was calculated by using the seed cotton yield obtained from net plot area and seed cotton weight of already separated 20 bolls was added in it. Seed cotton yield of each plot was converted to kg ha^-1^.

### Quality parameters

Staple length, a primary determinant of cotton quality, was measured according to the method of Krifa [20]. To determine the fiber elongation percentage, a method proposed by Hunt (1978) was followed. Fiber quality traits i.e. fiber strength and fiber maturity ratio were studied by putting a 20 (g) sample of lint in a latest computerized high Volume Instrument (HVI) USTER-900A in fiber testing laboratory, Fiber Technology Department, University of Agriculture Faisalabad. Fiber strength is the tensile strength of fiber which is measured in g/tex.

### Statistical analysis

The data collected were analyzed statistically by using Fisher’s analysis of variance technique. Data was normally distributed in most of the cases. While, in case of percentages data were subjected to arcsine transformation before the ANOVA. Difference among treatments’ means was compared using least significant difference test at 5% probability level [21].

## Results

Data of plant height showed significant differences among the tested cultivars and rates of K (K) during both years 2016 & 2017 (Table 2). Among cultivars of cotton, CYTO-301 produced significantly longer plants i.e. 134.75 cm at the highest K level than CYTO-142 and IUB-2013. Similarly, values for plant dry matter varied significantly in both cultivars and different K levels. Among cultivars, CYTO-301 produced more plant dry matter (959 and 1005) at highest level of K) followed by CYTO-142 and IUB-2013 in both years of experimentation at the highest level of K (120 kg K_2_O). Fruiting points data is shown in Table 3. Fruit points varied significantly among different levels of K application. The CYTO-301 depicted more fruiting points (461 and 453) followed by CYTO-142 and IUB-2013 at the highest K levels (120 kg K_2_O) during both years of study. Shedding percentage (%) also significantly improved by different fertilizer rates of K. In case of cotton cultivars, CYTO-301 showed significantly minimum shedding percentage (38% and 43%) than CYTO-142 and IUB-2013. Regarding fertilizer rates, maximum value of shedding percentage (%) was obtained where K fertilizer was not applied. Interactive effects of cultivars and K levels was also found significant regarding shedding percentage. Minimum shedding percentage (%) was recorded in CYTO-301 by K application at the rate of 120 kg per hectare.

**Table 2:** Impact of different levels of soil applied K_2_O on plant height and total plant dry matter of cotton cultivars grown under K deficient soil.

Parameters	Plant Height (cm)								Total Plant Dry Matter							
Years	2016				2017				2016				2017			
Cultivars	CYTO-301	CYTO-142	IUB-2013	Mean (T)	CYTO-301	CYTO-142	IUB-2013	Mean (T)	CYTO-301	CYTO-142	IUB-2013	Mean (T)	CYTO-301	CYTO-142	IUB-2013	Mean (T)
**0 kg K**_**2**_**O**	110.00	103.25	100.25	104.50 D	108.75	104.00	100.25	104.33 D	973 de	953 f	941 g	956 C	852 g	814 i	781 j	816 D
**40 kg K**_**2**_**O**	118.50	114.00	106.75	113.08 C	116.75	113.50	109.25	113.17 C	980 cd	974 de	962 e	972 B	896 e	871 f	836 h	862 C
**80 kg K**_**2**_**O**	128.75	123.50	116.50	122.92 B	127.25	124.25	118.50	123.33 B	995 ab	986 bc	976 cd	985 AB	932 bc	911 d	884 ef	909 B
**120 kg K**_**2**_**O**	134.75	128.75	124.50	129.33 A	134.25	128.50	124.50	129.08 A	1005 a	990 b	985 b-d	993 A	959 a	940 b	915 cd	938 A

C = Cultivars, T = Treatments, NS = Non-significant, Different alphabets with in the same column or row indicate statistically significant differences.

**Table 3:** Impact of different levels of soil applied K_2_O on fruiting points and shedding percentage of cotton cultivars grown under K deficient soil.

Parameters	Fruiting Points								Shedding percentage (%)							
Years	2016				2017				2016				2017			
Cultivars	CYTO-301	CYTO-142	IUB-2013	Mean (T)	CYTO-301	CYTO-142	IUB-2013	Mean (T)	CYTO-301	CYTO-142	IUB-2013	Mean (T)	CYTO-301	CYTO-142	IUB-2013	Mean (T)
**0 kg K**_**2**_**O**	415 e	411 ef	403 f	410 D	405 ef	402 ef	391 f	399 D	60 h	70 d	81 a	70 A	52 gh	65 de	74 a	64 A
**40 kg K**_**2**_**O**	428 d	423 de	421 de	424 C	419 d	413 de	408 e	413 C	56 i	67 e	78 b	67 B	47 i	63 e	72 b	61 B
**80 kg K**_**2**_**O**	445 b	439 c	431 cd	438 B	435 bc	430 c	423 cd	429 B	50 j	66 ef	77 bc	64 C	43 j	60 f	70 c	58 C
**120 kg K**_**2**_**O**	461 a	453 ab	449 b	454 A	453 a	447 ab	441 b	447 A	43 k	63 g	73 c	60 D	38 k	56 g	66 d	53 D

C = Cultivars, T = Treatments, NS = Non-significant, Different alphabets with in the same column or row indicate statistically significant differences.

There were significant differences among different treatments i.e. cultivars and different rates of K application regarding number of bolls (Table 4). It is obvious from the result that the boll number was increased by increasing K rate. The highest numbers of bolls (139, 135) were produced in case of CYTO-301 with K fertilizer at the rate of 120 kg per hectare during both the years of experimentation. Similar trend was also found regarding average boll weight (g) (Table 4). Different treatments i.e. cultivars and rates of K had significant effect on seed cotton yield (Table 5). Highest seed cotton yield of 2908 kg ha^-1^ and 2841 kg ha^-1^ was recorded in CYTO-301 cultivar. While, the lowest yield of 2379 kg ha^-1^ and 2361 kg ha^-1^ was produced in case of IUB-2013 during both years of experiments. While all the four different K fertilizer application rates showed statistically significant results. Highest seed cotton yield (2808 kg ha^-1^ and 2673 kg ha^-1^) was produced at the highest dose of K in both years. While the lowest seed cotton yield (2467 kg ha^-1^ and 2445 kg ha^-1^) was produced when no K was applied during both years.

**Table 4:** Impact of different levels of soil applied K_2_O on number of bolls per plant and boll weight of cotton cultivars grown under K deficient soil.

Parameters	Number of Bolls Plant^-1^								Boll Weight (g)							
Years	2016				2017				2016				2017			
Cultivars	CYTO-301	CYTO-142	IUB-2013	Mean (T)	CYTO-301	CYTO-142	IUB-2013	Mean (T)	CYTO-301	CYTO-142	IUB-2013	Mean (T)	CYTO-301	CYTO-142	IUB-2013	Mean (T)
**0 kg K**_**2**_**O**	130 cd	120 f	97 i	116 D	117 d	101 g	78 k	99 D	2.1 cd	1.8 cd	1.1 d	1.6 D	1.3 d	1.1 d	0.9 d	1.1 D
**40 kg K**_**2**_**O**	134 bc	125 e	108 h	122 C	124 c	107 f	84 j	105 C	3.1 bc	2.8 bc	2.6 c	2.8 C	3.1 bc	2.5 c	2.1 cd	2.6 C
**80 kg K**_**2**_**O**	136 b	128de	114 g	126 B	130 b	112 e	90 i	111 B	3.9 b	3.8 b	3.4 bc	3.7 B	3.6 b	3.4 bc	3.2 bc	3.4 B
**120 kg K**_**2**_**O**	139 a	131 c	118 fg	129 A	135 a	122 cd	96 h	118 A	5.2 a	4.5 ab	4.3 ab	4.7 A	4.8 a	4.5 ab	4.2 ab	4.5 A

C = Cultivars, T = Treatments, NS = Non-significant, Different alphabets with in the same column or row indicate statistically significant differences.

**Table 5:** Impact of different levels of soil applied K_2_O on seed cotton yield of cotton cultivars grown under K deficient soil.

Parameters	Seed Cotton Yield (kg ha^-1^)							
Years	2016				2017			
Cultivars	CYTO-301	CYTO-142	IUB-2013	Mean (T)	CYTO-301	CYTO-142	IUB-2013	Mean (T)
**0 kg K**_**2**_**O**	2653 de	2540 f	2208 i	2467 D	2695 de	2531 f	2118 i	2448 d
**40 kg K**_**2**_**O**	2814 c	2611 ef	2349 h	2591 C	2746 c	2622 ef	2368 h	2579 c
**80 kg K**_**2**_**O**	2988 b	2674 e	2421 g	2694 B	2871 b	2634 e	2435 g	2647 b
**120 kg K**_**2**_**O**	3175 a	2713 d	2537 fg	2808 A	3060 a	2704 d	2524 fg	2763 a

C = Cultivars, T = Treatments, NS = Non-significant, Different alphabets with in the same column or row indicate statistically significant differences.

Highest staple length was produced by cultivar CYTO-301 and the lowest staple length was produced by IUB-2013 cultivar during both the years of experiments (Table 6). The maximum staple length was calculated when highest dose of K was applied which reduced to minimum where K fertilizer was not applied. Similar observations were recorded regarding fiber elongation percentage (Table 6). The results indicated that different cultivars and rates of K fertilizer significantly influenced the fiber strength and fiber maturity ratio (Table 7). The highest fiber strength and maturity ratio were observed in CYTO-301 while the lowest fiber strength and maturity ratio were produced by IUB-2013 cultivar during both the years of experiment. Fiber strength and maturity ratio were produced with 120 kg of K per ha during both the year of experimentation while lowest values were observed where K was not applied.

**Table 6:** Impact of different levels of soil applied K_2_O on staple length and fiber elongation percentage of cotton cultivars grown under K deficient soil.

Parameters	Staple Length								Fiber Elongation Percentage (%)							
Years	2016				2017				2016				2017			
Cultivars	CYTO-301	CYTO-142	IUB-2013	Mean (T)	CYTO-301	CYTO-142	IUB-2013	Mean (T)	CYTO-301	CYTO-142	IUB-2013	Mean (T)	CYTO-301	CYTO-142	IUB-2013	Mean (T)
**0 kg K**_**2**_**O**	28	26	24	26 D	28	27	26	27.0 D	5.7	5.3	4.8	5.3 D	5.5	5.3	4.8	5.2 D
**40 kg K**_**2**_**O**	29	28	24	27 C	29	28	27	28.0 C	5.9	5.5	5.0	5.5 C	5.7	5.5	5.0	5.4 C
**80 kg K**_**2**_**O**	30	28	26	28 B	29	28	28	28.3 B	6.0	5.7	5.2	5.6 B	5.8	5.6	5.3	5.6 B
**120 kg K**_**2**_**O**	31	30	28	29.67 A	30	29	29	29.3 A	6.1	5.8	5.5	5.8 A	5.9	5.7	5.4	5.7 A

C = Cultivars, T = Treatments, NS = Non-significant, Different alphabets with in the same column or row indicate statistically significant differences.

**Table 7:** Impact of different levels of soil applied K_2_O on fiber strength and fiber maturity ratio of cotton cultivars grown under K deficient soil.

Parameters	Fiber Strength								Fiber Maturity Ratio							
Years	2016				2017				2016				2017			
Cultivars	CYTO-301	CYTO-142	IUB-2013	Mean (T)	CYTO-301	CYTO-142	IUB-2013	Mean (T)	CYTO-301	CYTO-142	IUB-2013	Mean (T)	CYTO-301	CYTO-142	IUB-2013	Mean (T)
**0 kg K**_**2**_**O**	94.6	90.2	89.5	91.5 D	90.4	89.5	88.4	89.4 D	1.15	1.11	1.00	1.08 C	1.11	1.07	0.98	1.05 B
**40 kg K**_**2**_**O**	95.2	91.2	90.4	92.3 C	91.0	91.4	90.3	90.9 C	1.16	1.12	1.04	1.11 B	1.12	1.08	1.00	1.06 B
**80 kg K**_**2**_**O**	95.7	93.2	91.0	93.3 B	93.5	92.6	91.5	92.5 A	1.16	1.13	1.05	1.11 B	1.12	1.08	1.01	1.07 B
**120 kg K**_**2**_**O**	96.2	94.5	92.2	94.3 A	94.3	93.1	92.4	93.3 A	1.17	1.13	1.08	1.13 A	1.15	1.10	1.04	1.10 A

C = Cultivars, T = Treatments, NS = Non-significant, Different alphabets with in the same column or row indicate statistically significant differences.

## Discussion

This experiment was accomplished in semi-arid environment of Multan region to explore the influences of K_2_O fertilizer on performance of cotton cultivars. All the levels of K fertilizer significantly (*P < 0*.*05*) improved the growth, yield and quality parameters of cotton cultivars. Doses in this experiment were set according to a preliminary trial and review of literature. In present study, maximum improvement in plant height was observed by application of K at 120 kg ha^-1^. The findings of current experimentation are in line with the findings of Akhter *et al*. [22] and Ali *et al*. [23] who reported that cotton cultivars are more receptive to K fertilizer regarding plant height. As a matter of fact, K has a main role in photosynthesis, water storage control and stomata opening in leaves [24]. Significant role of K has been established in root elongation and development of an effective root system which further aids plants to uptake nutrients. So, ideal plant height can be attributed to the optimum dose of K [18]. Significantly higher dry matter (959 and 1005%) was calculated at higher dose of K in our study. These findings are comparable to the findings of Hasanuzzaman et al. [24] who have reported positive correlation of K fertilizer with plant dry matter. It happened due to the fact that K has significant effect on total dry matter of cotton plants. Where, K deficiency can reduce partitioning to roots and inhibited leaf photosynthetic rates [25].

Significantly higher fruiting points (453 and 461) were observed at highest dose of K in cv. Cyto-301. Additionally, the boll weight (4.8 and 5.2g) was also enhanced at higher K level. These findings are comparable to the study of Read *et al*. [26] and Tariq *et al*. (2018) [9] who have reported that cotton yield was significantly enhanced by the K application. The enhanced number of fruiting points and boll weight can be attributed to higher K requirement during boll setting where bolls act as major sink. Contrarily, boll shedding was reduced [8, 9, 27, 28]. Also, the use of K in cotton improves water use efficiency thus surplus water pressure within the boll increases the weight of the boll [29].

The Cyto-301 showed significantly lower shedding percentage (38.00 and 43.00%) at the highest dose of K (120 kg ha^-1^). Results were comparable to the findings of study conducted by Rasool *et al*. [30]. Interestingly, when due to heavy load set the demand for K is increased and if the K is not supplied in enormous amount it results in the shedding of reproductive structures [31, 32].

Results accomplished that higher value for staple length (30 and 31) and fiber maturity parameters were attained in Cyto-301 at highest level of K. Findings regarding fiber elongation percentage were harmonized with the findings of Hallikeri *et al*. [33] and Ali *et al*. [15], who accomplished that increasing the quantity of inorganic fertilizer boosted the fiber elongation and fiber maturity traits. The K is involved protein production which enables the fiber to get more elongated [34]. The enhanced fiber maturity ratio indicated that diverse K use significantly exaggerated fiber maturity ratio because of varietal dissimilarities [35].

The improvement in cotton performance due to K application might be attributed to the increased photosynthetic rate owing to role of K in CO_2_ fixation and cell turgor control [36]. The K application in cotton is also believed to extend N absorption, which causes vigorous vegetative growth [15] and ultimately increases yield. The use of K fertilizers in cotton enhanced metabolic activity and improved staple length, tensile strength, fiber micronaire, and decreased the amount of damaged fiber [37]. Several other studies have reported an improvement in seed-cotton yield and fiber quality due to K application in cotton [22, 34, 38, 39].

## Conclusion

Application of K at 120 kg ha^-1^ produced maximum plant height, dry matter, fruiting point, bolls per plant, average boll weight and seed cotton yield with minimum shedding percentage. Fiber quality parameters including staple length, fiber strength and maturity ratio, and micronaire (fineness) were also significantly improved by application of K at 120 kg ha^-1^. Cultivar CYTO-301 was found more responsive to K fertilizer as compared to CYTO-142 and IUB-2013. Finding of present experimentation depicted that higher level of K fertilizer (120 kg ha^-1^) is considered appropriate to produce good quality of fiber with enhanced seed cotton yield. Future studies can work on doses higher than 120 Kg ha^-1^ along with different ecological conditions to further inquire the role of K in cotton growth, development and yield.
